# P-926. Defaults by Design: Impact of Default Antimicrobial Order Durations on Prescribing Patterns in the Emergency Department

**DOI:** 10.1093/ofid/ofaf695.1130

**Published:** 2026-01-11

**Authors:** Elisabeth Chandler, Ashley L Cubillos, Stephanie R Ducas, Joy Uzoma, Mary B Saunders

**Affiliations:** Gulf Coast Medical Center, Fort Myers, FL; Banner North Colorado Medical Center, Loveland, CO; NCH Healthcare System, Fort Myers, Florida; Lee Health, Fort myers, Florida; Lee Health, Fort myers, Florida

## Abstract

**Background:**

Many outpatient infections can be effectively treated with shorter (5-day) rather than longer (7-14 day) antimicrobial courses. While emergency department (ED)-focused antimicrobial stewardship (AMS) efforts are expanding, electronic health record (EHR) optimizations to impact prescribing of antimicrobial durations have not been robustly defined.

**Methods:**

This quality improvement initiative occurred at five EDs within a community health system. An update was made in the EHR to change the default dispense quantity of all oral antimicrobials at ED discharge to a 5-day supply. Adult patients receiving ≥1 oral antimicrobial at ED discharge were included. Pre- (2022) and post-intervention (2023) data were compared. The primary endpoint was the average antimicrobial days supply prescribed. Other endpoints included patient demographic and encounter data, primary diagnoses, antimicrobials prescribed, and 30-day ED readmission.

**Results:**

There were 33,226 ED encounters (39,253 antimicrobial prescriptions) pre-intervention vs. 37,545 post-intervention (44,824 antimicrobial prescriptions). Mean age was 49 years; 59% of patients were female. Top primary diagnoses were respiratory viruses, urinary tract infections, skin and soft tissue infections, intra-abdominal infections, and dental infections. Cephalosporins (34% vs. 36%) and penicillins (17% vs. 19%) were the most common antimicrobial classes prescribed in the pre- and post- groups, respectively. Average oral antibiotic days supply was reduced by 1 day pre-post intervention (7.9 days vs. 6.9 days; p < 0.001), resulting in an estimated 36,000 days of antibiotics saved over the 1-year timeframe. There were no differences in 30-day ED readmission (17.6% vs. 17.8%, p=0.37).

**Conclusion:**

This high impact, low resource EHR update to default dispense quantities for ED-discharge oral antimicrobial prescriptions was associated with a significant reduction in antimicrobial days’ supply with no difference in 30-day ED readmission. Similar interventions could be considered to help support appropriate antibiotic prescribing in community health system ED environments with limited AMS resources.

**Disclosures:**

All Authors: No reported disclosures

